# Differences in Breast Cancer Survival between Public and Private Care in New Zealand: Which Factors Contribute?

**DOI:** 10.1371/journal.pone.0153206

**Published:** 2016-04-07

**Authors:** Sandar Tin Tin, J. Mark Elwood, Ross Lawrenson, Ian Campbell, Vernon Harvey, Sanjeewa Seneviratne

**Affiliations:** 1 Section of Epidemiology and Biostatistics, School of Population Health, University of Auckland, Auckland, New Zealand; 2 Waikato Clinical School, University of Auckland, Hamilton, New Zealand; 3 Auckland District Health Board, Auckland, New Zealand; 4 Department of Surgery, University of Colombo, Colombo, Sri Lanka; ISPO, ITALY

## Abstract

**Background:**

Patients who received private health care appear to have better survival from breast cancer compared to those who received public care. This study investigated if this applied to New Zealand women and identified factors that could explain such disparities.

**Methods:**

This study involved all women who were diagnosed with primary breast cancer in two health regions in New Zealand, covering about 40% of the national population, between June 2000 and May 2013. Patients who received public care for primary treatment, mostly surgical treatment, were compared with those who received private care in terms of demographics, mode of presentation, disease factors, comorbidity index and treatment factors. Cox regression modelling was performed with stepwise adjustments, and hazards of breast cancer specific mortality associated with the type of health care received was assessed.

**Results:**

Of the 14,468 patients, 8,916 (61.6%) received public care. Compared to patients treated in private care facilities, they were older, more likely to be Māori, Pacifika or Asian and to reside in deprived neighbourhoods and rural areas, and less likely to be diagnosed with early staged cancer and to receive timely cancer treatments. They had a higher risk of mortality from breast cancer (hazard ratio: 1.95; 95% CI: 1.75, 2.17), of which 80% (95% CI: 63%, 100%) was explained by baseline differences, particularly related to ethnicity, stage at diagnosis and type of loco-regional therapy. After controlling for these demographic, disease and treatment factors, the risk of mortality was still 14% higher in the public sector patients.

**Conclusions:**

Ethnicity, stage at diagnosis and type of loco-regional therapy were the three key contributors to survival disparities between patients treated in public and private health care facilities in New Zealand. The findings underscore the need for more efforts to improve the quality, timeliness and equitability of public cancer care services.

## Introduction

Breast cancer is the most common cancer in women worldwide, accounting for 25% of all cancer cases and 15% of all cancer deaths [1]. In New Zealand (estimated female population of 2.2 million in 2011), there were 2867 new registrations (29% of all cancer registrations) and 636 deaths (15% of all cancer deaths) in 2011 [2]. While survival from breast cancer has improved over time, New Zealand has poorer net survival compared to some other developed nations [3, 4]. Additionally, breast cancer outcomes vary significantly within the country, with the worst outcome observed in Māori, Pacifika and people living in deprived areas [5].

Survival disparities observed across countries and within-country are likely to be due to variations in access to and quality of cancer care. New Zealand has a publicly funded national health system that provides free essential health care to all residents, regardless of insurance status. Alongside the public system, the private system provides a range of services including elective treatments and general surgical procedures which are mostly funded by private health insurance. The proportion of New Zealanders with private health insurance has been declining since 2008 and was about 30% in 2013 [6]. Overseas studies have linked private health care or insurance with earlier diagnoses, better treatments and higher survival outcomes for patients with breast cancer [7–13] and other malignancies [14–19] although some reported no significant association [20]. These findings, however, may not be directly applicable to the New Zealand context.

This paper therefore examined survival disparities in patients who received public vs. private care for their primary treatment in New Zealand and assessed the relative contribution of demographic, disease and treatment factors in explaining such disparities.

## Materials and Methods

### Study population

This study involved all women who were diagnosed with primary breast cancer in the Auckland and Waikato District Health Board Regions, where about two-fifths of the country’s population reside and which have similar incidence and mortality rates from breast cancer compared to the national figures [2], between June 2000 and May 2013.

### Data sources

The participants were identified from the Auckland and Waikato Breast Cancer Registers which are prospectively maintained population-based databases. From 2000 onward, the registers capture almost all newly diagnosed breast cancer cases in the respective district health board regions. The completeness of the Waikato Breast Cancer Register has been checked against the National Cancer Registry, and found to be 99% complete. The Auckland Breast Cancer Register is also complete since the removal of consent requirement in 2012 with 1% lost to follow up. Both databases contain more comprehensive and accurate information compared with national data sources [21–23].

Using the National Health Index (NHI) number, a unique identifier assigned to every person who uses health and disability support services in New Zealand, the registers are regularly linked to the National Cancer Registry and Mortality Collection. The New Zealand Cancer Registry contains information about all malignant tumours first diagnosed in New Zealand, except basal cell and squamous cell tumours of the skin. The Mortality Collection contains information about all deaths registered in the country [24]. The data were also linked to the National Minimum Dataset to obtain information on comorbidities. The National Minimum Dataset contains information about all day patients and inpatients discharged from all public hospitals and over 90% of private hospitals in New Zealand [25].

### Variables of interest

The exposure of interest in this analysis was the type of health care facility (public vs. private) where breast cancer was primarily treated (mostly surgical treatment), and the primary study outcome was breast cancer specific mortality. The categorisation of the death being due to breast cancer was made based on the medical records and death certificates. Information on the cause of death was ascertained by referring to original documents and cross-referencing with other national databases.

Other variables were selected based on their likely confounding or mediating effect on the exposure-outcome association, and include: patients’ demographics such as age, ethnicity and health domicile code, year of cancer diagnosis, mode of presentation (screen or symptomatic), tumour characteristics such as stage at diagnosis (Tumour, Node and Metastasis (TNM) system), grade, histological type and hormone receptor status, and treatments undertaken such as time to first treatment, loco-regional therapy (i.e., surgery and radiotherapy), chemotherapy and hormonal therapy. The health domicile codes represent patients’ usual residential address and categorised as urban (main urban, satellite urban and rural with high urban influence) and rural areas (others) based on Statistics New Zealand’s Urban/Rural Profile [26]. To assess the degree of neighbourhood deprivation, the domicile codes were also mapped on to the 2006 New Zealand Deprivation Index (NZDep) [27] with decile ten the most deprived and decile one the least. Patients’ comorbidity was measured using a C3 index score which is a cancer-specific index of comorbidity based on the presence of 42 chronic conditions recorded in the National Minimum Dataset for a period of five years prior to the diagnosis of cancer [28]. Each condition is weighed to its impact on one-year non-cancer mortality in a cancer cohort, and the weights are then summed to get a final comorbidity score.

### Analyses

Baseline data were presented as means with standard deviations and medians with interquartile ranges for continuous variables and percentages for categorical variables. All the data were complete for only 8505 participants (59.3%) as a large number of records, particularly prior to 2006, had missing data relating to HER-2 status. If HER-2 status was excluded, the data were complete for 75.0% of the participants. Missing values were computed using multiple imputation with ten complete datasets created by the Markov chain Monte Carlo method [29], incorporating all baseline co-variables and survival outcomes. Differences in baseline characteristics between patients who received public vs. private care were assessed using a two-sample T-test (for continuous variables) and Chi-squared test (for categorical variables) and adjusted for the year of diagnosis using PROC MIXED which fits a variety of mixed linear models to the data. Differences in the types of cancer treatment received were also adjusted for stage at diagnosis and biological factors.

To assess hazards of breast cancer specific mortality associated with the type of health care received, Cox proportional hazards regression modelling was performed with death from breast cancer as the failure variable, and death from another cause, or if alive, date of last follow-up, as censored observations. Hazard ratios (HR) were sequentially adjusted for five domains of co-variables: demographics, mode of presentation, disease factors, comorbidity index and treatment factors. When the continuous variables (age, time to first treatment and C3 index score) were added to the model, restricted cubic splines were used with knots at the 5th, 50th, and 95th percentiles [30]. Both age and menopausal status were retained in the models as R^2^ was 0.54 (equivalent to a variance inflation factor of 2.17) when the former was regressed on the latter. HER2 status was excluded as about one-third of the records had missing values and its impact on the exposure-outcome association was negligible.

The mediating role of each domain was determined by the percentage reduction in the β coefficient after inclusion of each domain in the model using the approach described previously [31]: 100 × (β_crude_-β_adjusted_)/β_crude_. The 95% confidence intervals relating to each percentage attenuation were estimated using a nonparametric bootstrapping method with 2000 re-samplings (with replacement). Subgroup analyses were undertaken by hormone receptor status. Sensitivity analysis was performed using total mortality (deaths from any cause) as the outcome variable. All analyses were performed using SAS (release 9.4, SAS Institute Inc., Cary, North Carolina).

### Ethics statement

This study extracted data on diagnosis, treatment and outcomes of patients diagnosed with breast cancer from the two registers, and linked the data to other national data collections such as the National Minimum Dataset and Mortality Collection. Written informed consent was not sought as it was not feasible to trace all patients. The data were analysed anonymously. Ethical approval for this study was obtained from the New Zealand Northern ‘A’ Ethics Committee (Ref. No. 12/NTA/42).

## Results

Of the 14468 patients who were diagnosed with primary breast cancer between June 2000 and May 2013, 8916 (61.6%) received public health care and 5553 (38.4%) received private health care (Table 1). Compared to the private sector patients, those who received public care were older, and more likely to be Māori, Pacifika or Asian and to reside in deprived neighbourhoods, rural areas and Waikato region (compared to Auckland).

**Table 1:** Baseline characteristics by health care facility type.

Characteristics		Public (N = 8916)		Private (N = 5553)		p-value	
		Crude	Adjusted^a^	Crude	Adjusted^a^	Crude	Adjusted^a^
**Age**	Mean (SD)	59.8 (14.1)	59.8	56.1 (11.9)	56.1	<0.0001	<0.0001
	Median (IQR)	59.0 (20.0)		55.0 (16.0)			
**Menopausal status**							
Pre-menopause	%	26.6	27.0	34.4	34.9	<0.0001	<0.0001
Peri-menopause	%	4.5	6.2	6.2	7.8		0.003
Post-menopause	%	66.0	66.8	56.8	57.4		<0.0001
*Missing/unknown*	%	*3*.*0*		*2*.*6*			
**Ethnicity**							
European	%	65.4	65.5	81.7	81.6	<0.0001	<0.0001
Māori	%	12.9	12.9	2.6	2.6		<0.0001
Pacifika	%	9.3	9.3	1.2	1.2		<0.0001
Asian	%	9.5	9.4	7.7	7.8		0.001
Other	%	2.9	3.0	6.8	6.7		<0.0001
**NZDep 2006**							
1–2	%	12.5	13.1	28.0	29.4	<0.0001	<0.0001
3–4	%	11.6	13.7	18.7	22.8		<0.0001
5–6	%	19.7	22.9	18.4	22.9		0.8
7–8	%	22.0	24.9	12.7	15.7		<0.0001
9–10	%	24.4	25.3	8.5	9.2		<0.0001
*Missing/unknown*	%	*10*.*0*		*13*.*8*			
**Area of residence**							
Urban	%	69.1	81.0	72.6	86.0	<0.0001	<0.0001
Rural	%	14.7	19.0	9.8	14.0		
*Missing/unknown*	%	*16*.*1*		*17*.*6*			
**Register**							
Auckland	%	74.5	74.5	84.3	84.3	<0.0001	<0.0001
Waikato	%	25.5	25.5	15.7	15.7		
**Screen-detected**							
Yes	%	42.6	42.3	46.7	47.1	<0.0001	<0.0001
No	%	57.5	57.7	53.3	53.0		
**Stage at diagnosis**							
0	%	13.4	13.4	15.9	15.9	<0.0001	<0.0001
I	%	35.1	35.0	40.5	40.5		<0.0001
II	%	31.7	31.8	31.3	31.4		0.6
III	%	14.3	14.4	10.6	10.6		<0.0001
IV	%	5.4	5.4	1.6	1.7		<0.0001
*Missing/unknown*	%	*0*.*0*		*0*.*0*			
**Grade**							
I	%	20.8	24.1	19.1	23.1	0.03	0.2
II	%	38.0	48.1	40.0	50.1		0.002
III	%	24.7	27.8	24.1	26.8		0.03
*Missing/unknown*	%	*16*.*5*		*16*.*8*			
**Histology**							
Ductal	%	69.2	73.0	67.4	73.1	<0.0001	0.5
Lobular	%	9.1	15.0	10.6	18.5		<0.0001
Other	%	11.3	12.1	7.6	8.4		<0.0001
*Missing/unknown*	%	*10*.*5*		*14*.*4*			
**ER/PR**							
ER+/PR+	%	56.5	59.1	54.8	57.9	0.2	0.1
ER+/PR-	%	12.8	19.2	12.5	19.3		0.5
ER-/PR+	%	1.2	5.1	1.3	5.5		0.5
ER-/PR-	%	15.8	16.7	16.6	17.2		0.3
*Missing/unknown*	%	*13*.*7*		*14*.*9*			
**HER-2**							
Positive	%	12.0	14.0	9.8	12.0	<0.0001	0.001
Equivocal	%	2.1	19.1	0.7	18.7		0.6
Negative	%	54.4	66.9	53.5	69.3		0.0003
*Missing/unknown*	%	*31*.*5*		*36*.*0*			
**C3 index scores**							
0	%	72.0	72.0	89.9	89.9	<0.0001	<0.0001
1	%	9.6	9.6	5.0	5.1		<0.0001
2	%	7.3	7.3	2.9	2.9		<0.0001
3+	%	11.2	11.2	2.2	2.1		<0.0001
**Time to first treatment (days)**	Mean (SD)	63.1 (191.3)	63.2	23.5 (87.8)	23.4	<0.0001	<0.0001
	Median (IQR)	34.0 (27.0)		15.0 (13.0)			
**Loco-regional therapy**							
Breast conserving surgery with radiotherapy	%	36.2	37.1^b^	49.4	47.8^b^	<0.0001	<0.0001^b^
Breast conserving surgery without radiotherapy	%	11.9	12.6^b^	12.9	11.7^b^		0.1^b^
Mastectomy with radiotherapy	%	15.7	14.6^b^	14.4	16.2^b^		0.006^b^
Mastectomy without radiotherapy	%	27.1	27.3^b^	22.1	21.7^b^		<0.0001^b^
No primary surgery	%	9.1	8.4^b^	1.3	2.5^b^		<0.0001^b^
**Chemotherapy**							
Yes	%	26.4	25.3^b^	31.3	32.9^b^	<0.0001	<0.0001^b^
No	%	73.7	74.7^b^	68.7	67.1^b^		
**Hormonal therapy**							
Yes	%	52.5	51.2^b^	51.1	53.3^b^	0.1	0.003^b^
No	%	47.5	48.8^b^	48.9	46.7^b^		
**Hormonal therapy**^c^							
Yes	%	70.5	60.3^d^	72.1	63.2^d^	0.1	0.0002^d^
No	%	29.5	39.7^d^	27.9	36.8^d^		

a Missing data imputed and proportion adjusted for the year of diagnosis

b Missing data imputed and proportion adjusted for the year of diagnosis and disease factors (stage at diagnosis, grade, histological type and ER/PR status)

c Restricted to hormone receptor positive patients

d Missing data imputed and adjusted for the year of diagnosis and disease factors (stage at diagnosis, grade and histological type)

Patients in the public sector were less likely to be diagnosed through screening compared to those in the private sector. They were also less likely to be diagnosed with early staged cancer (Stages 0-II) but tumour characteristics differed less significantly. They were more likely to have comorbidities. They had a (statistically) significantly longer time to the first treatment after diagnosis. They were less likely to receive breast conserving surgery with radiotherapy but more likely to have mastectomy without radiotherapy, or no primary surgery, even after adjusting for stage at diagnosis and other tumour factors. They were also less likely to receive chemotherapy and hormonal therapy.

**Fig 1:**
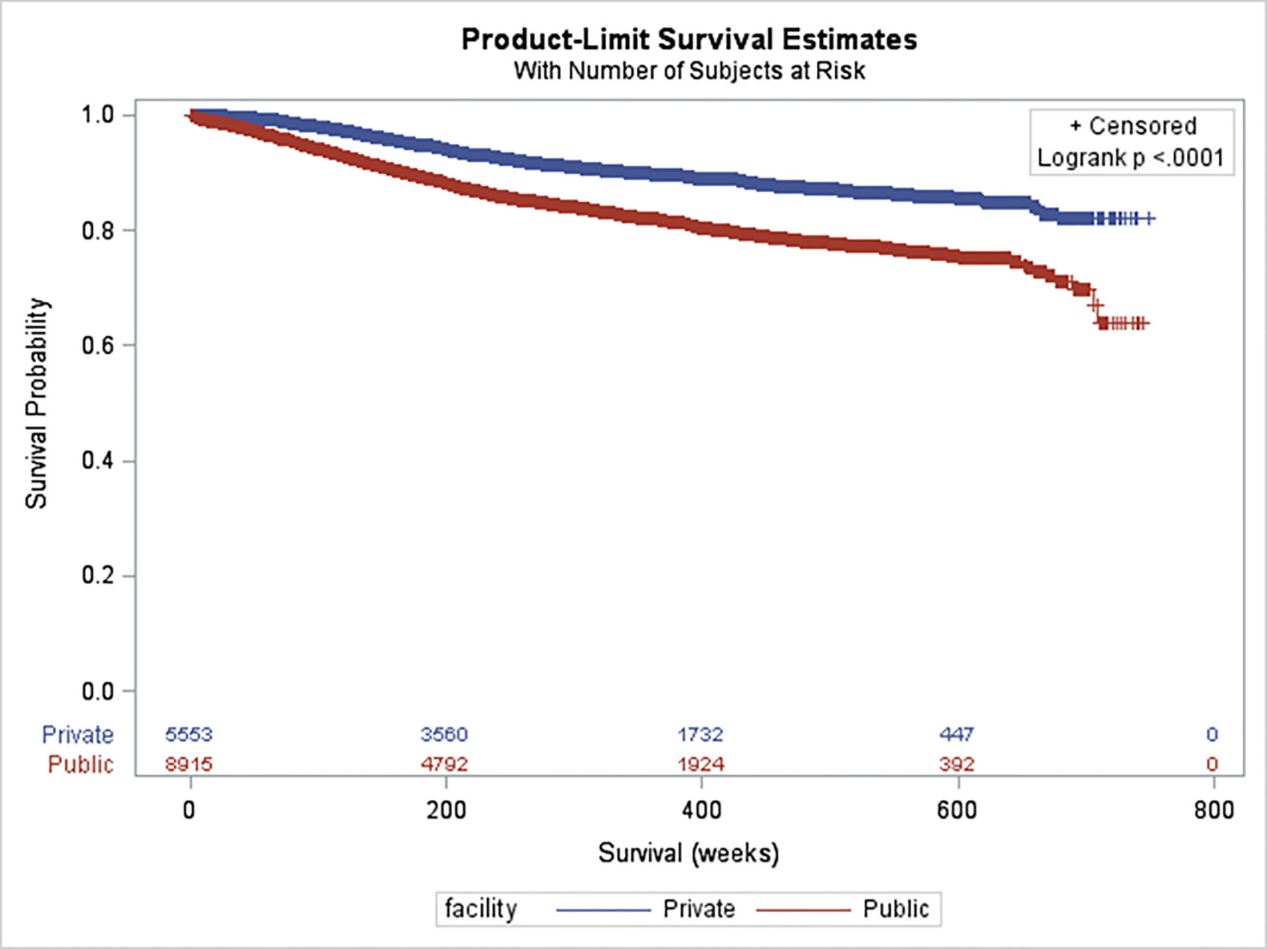
Breast cancer specific survival by health care facility type.

Patients who received public care had a (statistically) significantly higher risk of mortality from breast cancer (crude HR 1.95; 95% CI: 1.75, 2.17) (Fig 1 and Table 2). The crude HR was attenuated by 33% after controlling for differences in demographic factors but the mortality risk was still 56% higher in patients who received public care. Subsequent adjustment for mode of presentation slightly increased the risk. Adjustments for disease factors, comorbidity index and treatment factors resulted in a further 46%, 7% and 44% reduction in the HR. In particular, ethnicity, stage at diagnosis, and type of loco-regional therapy contributed most. Overall, factors included in the fully adjusted model accounted for 80.1% (95% CI: 62.6, 99.9) of the risk differential between public and private care. Yet, the risk of mortality was still 14% higher in the public sector patients. Similar results were observed if analyses were undertaken separately for hormone receptor positive and negative patients (S1 Table). Similar but slightly stronger associations were found in analyses using total mortality as the outcome variable (S2 Table).

**Table 2:** Hazards of death from breast cancer by health care facility type with stepwise adjustments.

Models	Additional variables in the model	Hazard ratios (95% CI)	% attenuation^a^ (95% CI^c^)	% attenuation^b^ (95% CI^c^)
1. Unadjusted		1.95 (1.75, 2.17)		
2. Model 1 + Demographics	Age, year of diagnosis	1.81 (1.63, 2.02)		
	Menopausal status	1.80 (1.61, 2.01)		
	Ethnicity	1.59 (1.42, 1.78)		
	NZDep2006	1.56 (1.39, 1.75)		
	Rurality	1.57 (1.39, 1.76)		
	Registers	1.56 (1.39, 1.76)	32.9 (24.6, 42.3)	32.9 (24.6, 42.3)
3. Model 2 + Detection method	Screen detected	1.59 (1.42, 1.79)	30.0 (21.3, 40.1)	-4.4 (-10.7, 1.3)
4. Model 3 + Disease factors	Stage	1.25 (1.11, 1.41)		
	Grade	1.27 (1.13, 1.43)		
	Histology	1.27 (1.13, 1.43)		
	ER/PR	1.29 (1.15, 1.45)	61.9 (48.4, 77.4)	45.5 (30.0, 64.6)
5. Model 4 + Comorbidity	C3 index scores	1.27 (1.12, 1.43)	64.5 (50.6, 80.4)	6.9 (0.9, 16.9)
6. Model 5 + Treatment factors	Time to first treatment	1.28 (1.13, 1.46)		
	Locoregional therapy	1.13 (0.99, 1.28)		
	Chemotherapy	1.14 (1.00, 1.29)		
	Hormonal therapy	1.14 (1.00, 1.30)	80.1 (62.6, 99.9)	43.9 (14.4, 98.8)

a % attenuation compared with Model 1

b % attenuation compared with the previous model

c 95% bootstrap confidence interval

## Discussion

### Main findings

In this study, about 62% of patients received public health care and they had a 95% higher average risk of mortality compared to those who received private care. There were significant differences in demographics, mode of presentation, disease factors, comorbidity index and treatment factors between the two populations, which explained approximately 80% of the survival disparities. The three key contributors were ethnicity, stage at diagnosis and type of loco-regional therapy.

### Strengths and limitations

This study used the data from two prospectively maintained population-based databases which contain comprehensive and near complete information about patients diagnosed with primary breast cancer. Linkage to the national databases also enabled us to ascertain information on cause of death and to obtain information on comorbidities. Yet, a considerable proportion of patients had missing data particularly relating to HER-2 status, most of whom were diagnosed prior to 2006 when HER-2 testing was not routine in New Zealand. We excluded this variable from the analyses as its impact on the exposure-outcome association was negligible. It was also not possible to assess the impact of some important factors such as smoking as such information was not recorded in the databases. NZDep2006 used in this analysis measures area-level deprivation and may not reflect an individual’s actual socioeconomic status although it has been validated previously [32]. Additionally, some patients treated in the private sector may have been transferred to a public facility for further care after their primary treatment. This may result in misclassification of exposure and bias the association estimates. Potential misclassification of cancer-specific deaths may also occur but such errors are likely to be similar in any subgroups being compared, and will only act to reduce observed differences to a small extent.

### Interpretations

A higher risk of mortality in patients who received public care can be partly explained by demographic differences in health care access. Overseas studies reported that access to private care was less common in ethnic minorities and socioeconomically disadvantaged groups [9, 12–16]. Similar findings were observed in this study. In particular, ethnic variations appear to be a major contributor to survival disparities. It is well documented that Māori and Pacifika women have poorer survival from breast cancer compared to other ethnic groups, which is mostly contributed by differential access to cancer care services [5, 33–35]. In a recent population-based case-control study involving about 1800 patients, a higher proportion of Maori and Pacifika women reported barriers to and delays in access to care compared to other ethnic groups with the most commonly reported barriers being “cost” and “fear” [36]. This underscores the need for more efforts to improve affordable and culturally sensitive cancer care services for Maori and Pacifika patients. Such efforts should be focused on the quality and timeliness of public health services where these patients are more likely to be treated.

After adjusting for demographic factors, patients in the public sector still had a 56% higher risk of mortality from breast cancer compared to those in the private sector. This could be due to deficits in the public health care system along the cancer care pathway. Consistent with previous research [8–18], our analysis found that patients who received public care were less likely to be diagnosed with early staged cancer and to receive timely cancer treatments.

Stage at diagnosis is an indicator of health care access and is a key contributor to survival disparities [33, 37]. Likewise in this study, late diagnosis has contributed significantly to survival differences between private and public care, and could be partly explained by differential access to screening services–the proportion of patients with screen-detected breast cancer was lower in the public sector than the private sector. New Zealand has a national breast cancer screening program established in 1998, which initially offered publicly funded mammography to all asymptomatic women aged 50 to 64 years and was extended to include women aged 45 to 49 years and 65 to 69 years in 2004. While screening coverage has improved over time and met the target of 70%, coverage for Māori women is relatively low [38], and there is room for improvement. Other factors that could contribute to late diagnosis include patient factors such as health literacy, low socioeconomic status, fear of cancer and other psychosocial factors, and health system factors such as those related to primary care practices and practitioners [39–42], and are worthy of further investigation.

In addition to experiencing diagnosis delays, patients treated in the public sector had a significantly longer time (40 more days on average) to the first treatment after diagnosis as reported previously [43]; however, treatment delays have not accounted substantially for their inferior survival. A more important contributing factor is differences in loco-regional therapy received. While some patients in the public sector may have been treated by less experienced surgeons with lower case loads, which has been associated with poorer outcomes in previous overseas studies [44, 45], omission of definitive treatment is much more likely to be the cause. We found that patients in the public sector were less likely to receive primary surgery or radiotherapy. This is not surprising as the private sector provides quicker access to elective surgery and other health services which are mostly funded by health insurance. We also found a lower rate of breast conserving surgery in patients treated in the public sector as reported in some US studies [11, 46]. This may reflect a higher prevalence of advanced cancer in the public sector but our analysis accounted for stage at diagnosis and biological factors. Another possible explanation is patient preference. A previous Australian study reported that patients in the public sector were less likely to accept the recommended standard chemotherapy [47] but little is known with regard to loco-regional treatments in breast cancer patients.

In New Zealand, some initiatives are in place to improve cancer care and support for patients and their families, for example, the Faster Cancer Treatment (FCT) program established by the Ministry of Health [48]. However, survival disparities observed in this analysis as well as in previous research suggest that a lot more needs to be done to improve the quality, timeliness and equitability of public cancer care services.

## Conclusions

The risk of breast cancer specific mortality was 95% higher in patients who received public health care compared to those who received private care. About 80% of this survival disparity could be explained by differences specifically examined in the study, particularly related to ethnicity, stage at diagnosis and type of loco-regional therapy. After accounting for these demographic, disease and treatment factors, the risk of mortality was still 14% higher in the public sector patients. The findings underscore the need for more efforts to improve the quality, timeliness and equitability of public cancer care services in New Zealand.

## Supporting Information

S1 Table Hazards of death from breast cancer in hormone receptor positive and negative patients by health care facility type with stepwise adjustments. (DOCX)

S2 Table Hazards of death from any cause by health care facility type with stepwise adjustments. (DOCX)
