# Supplementary material for: Differences in Breast Cancer Survival between Public and Private Care in New Zealand: Which Factors Contribute?
Source: PLoS One. 2016 Apr 7;11(4):e0153206. doi: 10.1371/journal.pone.0153206 (PMC4824501; doi:10.1371/journal.pone.0153206)
Supplement: S1 Table — (DOCX) [file pone.0153206.s001.docx]

**Table S1. Hazards of death from breast cancer in hormone receptor positive and negative patients by health care facility type with stepwise adjustments**

| **Models** | **Additional variables in the model** | **Hormone receptor positive patients** | | | **Hormone receptor negative patients** | | |
| --- | --- | --- | --- | --- | --- | --- | --- |
|  |  | **Hazard ratio (95% CI)** | **% attenuation^a^** | **% attenuation^b^** | **Hazard ratio (9%% CI)** | **% attenuation^a^** | **% attenuation^b^** |
| 1. Unadjusted |  | 2.22 (1.93, 2.55) |  |  | 1.61 (1.35, 1.92) |  |  |
| 2. Model 1 + Demographics | Age, year of diagnosis | 2.02 (1.75, 2.32) |  |  | 1.53 (1.27, 1.83) |  |  |
|  | Menopausal status | 2.00 (1.74, 2.31) |  |  | 1.52 (1.27, 1.82) |  |  |
|  | Ethnicity | 1.73 (1.49, 2.00) |  |  | 1.42 (1.17, 1.71) |  |  |
|  | NZDep2006 | 1.69 (1.45, 1.96) |  |  | 1.40 (1.16, 1.70) |  |  |
|  | Rurality | 1.69 (1.46, 1.96) |  |  | 1.41 (1.16, 1.71) |  |  |
|  | Registries | 1.68 (1.45, 1.95) | 34.6 |  | 1.41 (1.16, 1.71) | 27.7 |  |
| 3. Model 2 + Detection method | Screen detected | 1.70 (1.47, 1.97) | 33.4 | -1.9 | 1.46 (1.21, 1.77) | 20.0 | -10.8 |
| 4. Model 3 + Disease factors | Stage | 1.35 (1.16, 1.56) |  |  | 1.15 (0.95, 1.40) |  |  |
|  | Grade | 1.37 (1.18, 1.59) |  |  | 1.16 (0.96, 1.41) |  |  |
|  | Histology | 1.37 (1.18, 1.59) | 60.7 | 41.0 | 1.17 (0.96, 1.42) | 67.4 | 59.3 |
| 5. Model 4 + Comorbidity | C3 index scores | 1.34 (1.15, 1.56) | 63.4 | 6.9 | 1.15 (0.94, 1.40) | 71.3 | 11.9 |
| 6. Model 5 + Treatment factors | Time to first treatment | 1.33 (1.14, 1.57) |  |  | 1.19 (0.96, 1.47) |  |  |
|  | Locoregional therapy | 1.14 (0.97, 1.34) |  |  | 1.15 (0.93, 1.43) |  |  |
|  | Chemotherapy | 1.14 (0.97, 1.35) |  |  | 1.16 (0.94, 1.44) |  |  |
|  | Hormonal therapy | 1.16 (0.98, 1.37) | 81.4 | 49.2 | 1.15 (0.93, 1.43) | 70.6 | -2.6 |

a % attenuation compared with Model 1

b % attenuation compared with the previous model
