# Supplementary material for: Differences in Breast Cancer Survival between Public and Private Care in New Zealand: Which Factors Contribute?
Source: PLoS One. 2016 Apr 7;11(4):e0153206. doi: 10.1371/journal.pone.0153206 (PMC4824501; doi:10.1371/journal.pone.0153206)
Supplement: S2 Table — (DOCX) [file pone.0153206.s002.docx]

**Table S2. Hazards of death from any cause by health care facility type with stepwise adjustments**

| **Models** | **Additional variables in the model** | **Hazard ratios (95% CI)** | **% attenuation^a^** | **% attenuation^b^** |
| --- | --- | --- | --- | --- |
| 1. Unadjusted |  | 2.35 (2.16, 2.56) |  |  |
| 2. Model 1 + Demographics | Age, year of diagnosis | 1.86 (1.71, 2.02) |  |  |
|  | Menopausal status | 1.84 (1.69, 2.01) |  |  |
|  | Ethnicity | 1.64 (1.50, 1.79) |  |  |
|  | NZDep2006 | 1.63 (1.49, 1.79) |  |  |
|  | Rurality | 1.64 (1.49, 1.79) |  |  |
|  | Registries | 1.63 (1.49, 1.79) | 42.6 | 42.6 |
| 3. Model 2 + Detection method | Screen detected | 1.64 (1.50, 1.80) | 42.1 | -0.8 |
| 4. Model 3 + Disease factors | Stage | 1.42 (1.30, 1.56) |  |  |
|  | Grade | 1.43 (1.31, 1.57) |  |  |
|  | Histology | 1.43 (1.30, 1.56) |  |  |
|  | ER/PR | 1.44 (1.31, 1.57) | 57.5 | 26.5 |
| 5. Model 4 + Comorbidity | C3 index scores | 1.34 (1.22, 1.47) | 65.6 | 19.2 |
| 6. Model 5 + Treatment factors | Time to first treatment | 1.31 (1.19, 1.45) |  |  |
|  | Locoregional therapy | 1.15 (1.04, 1.27) |  |  |
|  | Chemotherapy | 1.15 (1.04, 1.27) |  |  |
|  | Hormonal therapy | 1.17 (1.06, 1.29) | 81.4 | 46.0 |

a % attenuation compared with Model 1

b % attenuation compared with the previous model
